# Supplementary material for: Identification of Freezing-Responsive microRNAs and Their Targets in Chinese Jujube by Small RNA and Degradome Sequencing
Source: Int J Mol Sci. 2026 Sep 4;27(17):7912. doi: 10.3390/ijms27177912 (PMC13566890; doi:10.3390/ijms27177912)
Supplement: Supplementary file 1 [file ijms-27-07912-s001.zip › Supplementary Material S2/novel_miR_180.pdf]

Provisional ID : novel_miR_180
Score total : 24.4
Score for star read(s) : 3.9
Score for read counts : 22.6
Score for mfe : 0.1
Score for randfold : -2.2
Score for cons. seed :
Total read count : 56
Mature read count : 55
Loop read count : 0
Star read count : 1

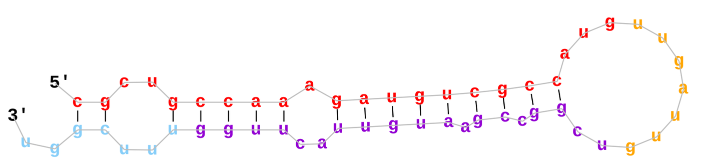

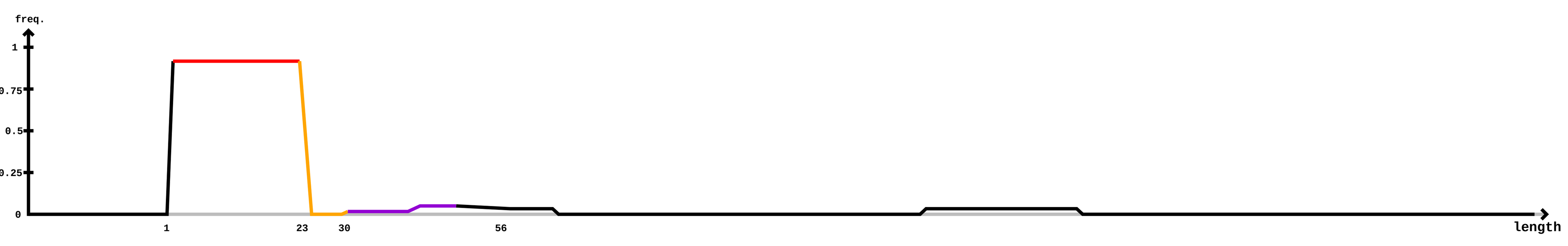


```
                      Mature                 Star
5'- gcuccggagcugucaucaaugccgcugccaaagaugucgccauguugauugucggccgaauguuacuugguuucgguauuggguucaccaaucagguuugaaucucucucucucucucucccucucccccucgcccucucuuuuauauauggaucuuggacgucaauuugucugguggguuuuguuuuuguuuuugguuuggaucgagaauauauauauauauauuaauuuuuugggugugucugccu -3' obs
    gcuccggagcugucaucaaugccgcugccaaagaugucgccauguugauugucggccgaauguuacuugguuucgguauuggguucaccaaucagguuugaaucucucucucucucucucccucucccccucgcccucucuuuuauauauggaucuuggacgucaauuugucugguggguuuuguuuuuguuuuugguuuggaucgagaauauauauauauauauuaauuuuuugggugugucugccu      exp
    .....((((.......((((((((..(((((.(((((((((...........)).)).)))))..)))))..))))))))(((((((.....)))..))))).............))))..........(((((.......(((((((((((((.(((((......)))))...))))).(((...((((((((((....))))))))))...))))))))))).........))))).........  reads  mm  sample
    ......................cgcugccaaagaugucgcAaug............................................................................................................................................................................................................  1  1  LCK-2
    ......................cgcugccaaagaugucgccaug............................................................................................................................................................................................................  31  0  LCK-1
    ......................cgcugccaaagaugucgccaug............................................................................................................................................................................................................  23  0  LCK-2
    ...................................................ucggccgaauguuacuugg..................................................................................................................................................................................  1  0  LT-1
    ..............................................................uuacuugguuucgguauuggguuc..................................................................................................................................................................  1  0  LT-3
    ...............................................................uacuugguuucgguauuggguuc..................................................................................................................................................................  1  0  LT-3
    ...................................................................................................................................................uauggaucuuggacgucaauuugucu...........................................................................  2  0  JCK-3
```
